# When genetics prevail: brain stimulation fails to overcome learning deficits from brain-derived neurotrophic factor *Val66Met*

**DOI:** 10.1016/j.cnp.2025.08.009

**Published:** 2025-09-04

**Authors:** Perianen Ramasawmy, Krisztián Iszak, Steffen Brüshaber, Viktória Kállay, Géza Gergely Ambrus, Walter Paulus, Andrea Antal

**Affiliations:** a Non-Invasive Brain Stimulation Lab, Department of Neurology, University Medical Center Göttingen, Georg-August University, Göttingen, Germany; b Department of Cardiology, Pneumology and Angiology, University Hospital Düsseldorf, Düsseldorf, Germany; c Universitätsklinik für Psychiatrie und Psychotherapie, Evangelisches Klinikum Bethel, Universitätsklinikum OWL der Universität Bielefeld, Bielefeld, Germany; d Department of Psychology, Bournemouth University, Poole, UK; e Department of Neurology, University Hospital of Munich, Ludwig-Maximilians-University, Munich, Germany

**Keywords:** tDCS, BDNF, Val66Met, transcranial stimulation, cognitive enhancement

## Abstract

**Background:**

The dorsolateral prefrontal cortex (DLPFC) is a key contributor to memory categorization. Brain-Derived Neurotrophic Factor (BDNF) *Val66Met* polymorphism affects the efficacy of neuronal plasticity induction. We investigated whether DLPFC-transcranial direct current stimulation (tDCS) influences categorization performance, and whether BNDF genotype modulates this effect.

**Methods:**

Sixty-two healthy individuals were randomized to receive 10 min of either anodal right DLPFC-tDCS at 1 mA, or sham tDCS during the training phase of a prototype distortion task. Categorization performance was assessed during, shortly after, and the morning following the stimulation. *Val66Met* polymorphism status was determined through BNDF genotyping.

**Results:**

*Val66Met* carriers showed poorer categorization ability than *Val/Val* homozygotes (p < 0.0001). tDCS did not enhance categorization performance overall, or reduce genotype-associated differences with these specific stimulation parameters. However, tDCS extinguished the prototype effect of the categorization task observed in our sample.

**Conclusions:**

Our findings underscore the importance of BDNF polymorphism in category learning. 1 mA anodal right DLPFC over the right DLPFC does not improve performance or offset genotype differences under tested conditions.

**Significance:**

BDNF *Val66Met* polymorphism influences category learning, and low-intensity tDCS does not counteract this effect, emphasizing the need to refine stimulation protocols for genotype-specific cognitive enhancement.

## Highlights

• *Met* carriers showed lower categorization ability than *Val/Val* homozygotes.
• Transcranial direct current stimulation(tDCS) did not improve categorization level.
• No difference in tDCS effects on categorization between carriers and homozygotes.

## 1 Introduction

From a cow categorizing a plant as either edible or poisonous, to humans categorizing an individual as either an enemy or a friend, category learning is crucial for the survival of any organism. It is the ability to perceive the higher-level structure of experiences and the similarities across particular experiences which enable us to group them into meaningful concepts and categories (Ell and Zilioli, 2012, Seger and Miller, 2010). Category learning in humans is believed to constitute a multi-system approach, with an explicit rule-based analytic categorization system which is different from the implicit and non-analytic learning system (Ashby et al., 1998, Ashby and Waldron, 1999, Poldrack and Foerde, 2008, Smith et al., 2012).

Prototype category learning is a type of categorization, whereby the knowledge of the category is still acquired even when contents within a category only share loose commonalities and no feature is prominent enough to be characterized as a verbal rule (Wu and Fu, 2021). The two prototype distortion tasks most commonly used to study category learning are the A/B and the A/not-A tasks (Ashby and Maddox, 2005, Ashby and Maddox, 2011, Bowman et al., 2020, Lei et al., 2010, Wirsich et al., 2014). The A/B prototype distortion task usually begins with a training phase, where subjects learn to actively allocate items—which does not include the prototype—via trial-and-error to one or more categories by presenting category labels. During the test phase, they are then asked to categorize the unlabeled stimuli, which included the prototypes, a set of novel stimuli and previously presented stimuli, into categories A or B. In most of the studies, participants are not provided with any feedback during the test phase of the task. A/B prototype category learning has been shown to be mediated by the explicit memory system, since individuals with impaired declarative memory demonstrated deficits in the A/B version of the prototype task (Wu and Fu, 2021, Zeithamova et al., 2008), with studies conducted in patients with amnesia (Knowlton and Squire, 1993), autism spectrum disorders (Vanpaemel and Bayer, 2021), Alzheimer’s disease (Bozoki et al., 2006, Heindel et al., 2013, Nosofsky et al., 2012), and Parkinson’s disease (Paul J. Reber and Squire, 1999).

Transcranial direct current stimulation (tDCS) has proven to be a promising non-invasive brain stimulation tool for cognitive enhancement, including categorization, in both healthy individuals and patients with cognitive or behavioral disorders (Antal et al., 2022, Begemann et al., 2020, Chen et al., 2022, Ciullo et al., 2021, Ferrucci et al., 2008, Jacobson et al., 2012). tDCS is a safe neurophysiological technique involving the delivery of a weak direct electric current to the scalp via surface electrodes (Nitsche and Paulus, 2000, Paulus, 2011). tDCS can induce changes in the cortical excitability both at the stimulation site and at the level of brain networks (Opitz et al., 2016, Radman et al., 2009) through modulating the neuronal membrane potential. Although many previous studies examined the effects of tDCS on categorization learning, their findings were inconsistent. In our previous study (Ambrus et al., 2011), we demonstrated that anodal tDCS of 1 mA applied over either the left or right dorsolateral prefrontal cortex (DLPFC) during an A/not-A variant of a prototype distortion task significantly impaired the ability of correctly categorizing prototypes compared to sham stimulation in healthy participants. Lupyan et al. (2012) showed that stimulation of the left inferior prefrontal cortex at 1.5 mA during a simple explicit categorization task altered the performance of healthy participants, with differential effects of cathodal and anodal tDCS. Moreover, a study testing the effects of anodal tDCS over the right cerebellum at 1.5 mA failed to demonstrate any enhancement in performance of healthy participants in an implicit categorization task (Verhage et al., 2017). Recently, healthy participants who received anodal or cathodal tDCS of the right ventrolateral prefrontal cortex at 2 mA showed higher performances on a novel categorization task, which involved classification of pictures of European streets, compared to sham stimulation (Gibson et al., 2020). The varying stimulation target and differences in the categorization task used in the studies could have resulted in the differential impacts of tDCS on category learning.

Previous imaging studies identified the frontal cortical areas as major players in prototype category learning (Ashby and Maddox, 2005, Ashby and Maddox, 2011, Bowman et al., 2020, Reber et al., 1998, Seger et al., 2000, Vogels et al., 2002). In this study, we target the right dorsolateral prefrontal cortex (DLPFC) as the study by Seger et al. (2000) found that this area is active during the whole duration of the acquisition phase of the A/B prototype distortion task, irrespective of the performance level. Moreover, A/B variant task exhibits greater activity in the frontal cortices than A/not-A variant which involves to a larger degree the posterior cortices and striatum (Zeithamova et al., 2008).

Brain-derived neurotrophic factor (BDNF) plays a key role in neuroplastic changes associated with learning and memory (Egan et al., 2003, Kowiański et al., 2017, Messaoudi et al., 2002). BDNF *Val66Met* polymorphism involves a single nucleotide polymorphism, whereby a *Val* is substituted by a *Met* at codon 66 in the 5′ pro-domain of the BDNF protein (Egan et al., 2003) approximately 40 % of studied German cohorts are *Met* carriers (Gajewski et al., 2011, Schüle et al., 2006). BDNF *Val66Met* polymorphism has shown minimal or no effects on implicit learning (Freundlieb et al., 2012, Veronica Witte et al., 2012) or working memory performance (Banner et al., 2011, Nagel et al., 2008, van den Bosch et al., 2021) compared to monozygotes. On the other hand, this SNP has been repeatedly demonstrated to have an influence on hippocampus-dependent declarative memory (Canivet et al., 2015, Kambeitz et al., 2012, Mandolini et al., 2019, Rovný et al., 2023) with *Met* carrier participants usually performing worse than *Val/Val* homozygotes, to which categorization performance has also been linked (Tunney and Fernie, 2012). Furthermore, studies have demonstrated the modulating impact of this SNP on the efficacy of cortical plasticity induced by non-invasive brain stimulation, with techniques ranging from tDCS (Antal et al., 2010, Puri et al., 2015), paired associated stimulation (Cheeran et al., 2008, Cirillo et al., 2012, Missitzi et al., 2011), and iTBS (Cheeran et al., 2008, Marsili et al., 2017). The results pointed towards a higher cortical excitability following stimulation in *Val* homozygotes than *Met* carriers in all the above-mentioned studies, except that prior studies including anodal tDCS of the primary motor cortex (M1) found higher cortical excitability in *Met* carriers than homozygotes (Antal et al., 2010, Puri et al., 2015). It is important to note that all these studies examine the cortical excitability of the M1.

Our investigation assessed the effects of anodal tDCS over the right DLPFC to categorization performance using a feedback-aided A/B variant of a visual prototype distortion task (Fried and Holyoak, 1984, Seger et al., 2000) in a parallel group design regarding the stimulation status. To our knowledge, this is the first study to research the effects of tDCS using the A/B variant of the prototype distortion task on category learning. Unlike the effects of the A/not-A variant of the prototype distortion task (Ambrus et al., 2011), we hypothesized that anodal stimulation will enhance the performance compared to sham stimulation. Based on previous research, individuals carrying the *Met* allele were expected to show lower categorization performance compared to *Val* homozygotes. Therefore, another aim of this study was to examine whether anodal tDCS leads to different levels of improvement in categorization between *Val* homozygotes and *Met*-carriers.

## 2 Materials and methods

### 2.1 Study design

We conducted a monocenter, randomized, parallel, double-blinded and sham-controlled study at the University Medical Center in Göttingen, Germany. The protocol was approved by the Ethics Committee of the University of Göttingen under the registration number 12/4/12, according to the 1964 Declaration of Helsinki and its revisions.

**Fig. 1:**
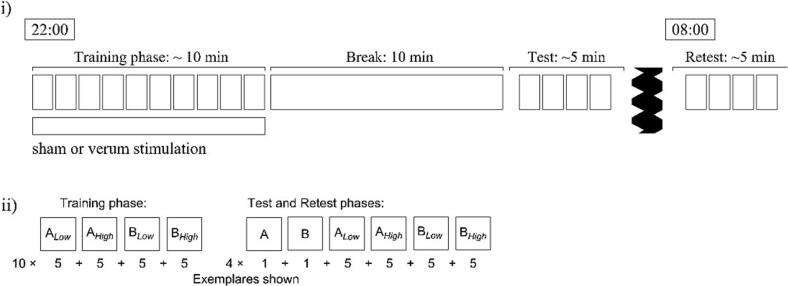
Study design. (i) In the evening, ten blocks (T1-T10) of feedback-aided training (with tDCS stimulation or sham, depending on the experimental group) were followed by a four-block test phase (E1-E4) without feedback after an unfilled break of 10 min. The participants returned the next morning to complete a second four-block, no-feedback test phase (M1-M4). (ii) During the training phase, with the aid of immediate feedback (indicating correct or incorrect response), subjects had to learn to categorize low- and high distortion versions of two prototype patterns. In the evening and morning test phases, the participants' categorization performance was tested on low- and high distortion patterns, as well as on the prototypes themselves.

Participants visited the medical center four times during the study. At the first visit, the participant was informed about the study, followed by a short neurological examination by a medical doctor. The participant’s consent was obtained for stimulation as well as for blood sampling for genetic testing. Following successful inclusion, participants were randomly allocated to either the anodal tDCS stimulation group, which received 10 min of anodal tDCS over the right DLPFC at 1 mA, or the sham group. The second visit began at 21:30 or 22:30, starting with 10 min of training phase for the A/B prototype distortion task concurrently paired with the respective stimulation protocol. After a 10 min break, a test phase was conducted for assessing categorization performance. At 07:30 or 08:30 the next day (depending on the time of the previous visit respectively), participants came for their third session, where their categorization ability was tested again. In the fourth visit, a whole blood sample of 7.5 mL was taken from participants by a study nurse and human genetic testing was conducted by the Institute of Human Genetics at the University Medical Center Göttingen. The study design is illustrated in Fig. 1. The study was double-blinded, meaning that both the participant and the experimenter were blinded to the tDCS protocol (sham or anodal tDCS) and BDNF-Genotype since the blood results were only available after the test phase. Analysis was also conducted in a blinded fashion.

### 2.2 Participants

Healthy individuals aged from 18 to 60 years were recruited via advertisements at the University of Göttingen. Efforts were made to ensure consistent participant inclusion and minimal waiting time for participation. The exclusion criteria for the study were as follows: history or presence of neurological and psychiatric diseases, diagnosis of major cardiovascular, pulmonary or musculoskeletal disorders, drug or alcohol abuse, presence of implanted metal devices in the head, neck or chest area, pregnancy or breastfeeding, consumption of chronic or acute medication targeting the nervous system at the time of the study, participation in another scientific or clinical study within 8 weeks prior to study inclusion. All participants had to have normal or corrected to normal visual acuity. Furthermore, it was mandatory that each individual’s first language be German. The participants were asked not to consume any alcoholic drinks or substances containing caffeine on the days of the experiment.

### 2.3 Transcranial direct current stimulation

tDCS was delivered by a battery-driven NeuroConn DC-Stimulator Plus stimulator (neuroConn GmbH, Ilmenau, Germany) through a pair of rubber standard rubber electrodes (3 cm × 3.5 cm;10.5 cm^2^), which were placed in rectangular sponges (5 cm × 7 cm; 35 cm^2^) soaked in isotonic sodium chloride solution. The anode was placed over the right DLPFC, while the cathode was positioned at C_z_ position of the 10/20 EEG system. The anodal position was standard for all participants at 9 cm to the right and 7 cm anterior relative to C_z_, similar to our previous study (Ambrus et al., 2011). Elastic rubber bands were used to secure the electrodes to the head during the stimulation. For the anodal tDCS stimulation, a constant current of 1 mA was delivered for 10 min with 20 s ramp up at the start and 10 s ramp down at the end of the stimulation. For the sham protocol, the electrode size and montage were identical to anodal tDCS and comprised an initial 20 s ramp-up from 0 to 1 mA, followed by 30 s tDCS at 1 mA and then a 10 s ramp-down to 0 mA. This sham protocol was validated and reproduced the brief tingling sensation under the electrodes similar to anodal stimulation (Ambrus et al., 2011, Ambrus et al., 2012).

### 2.4 Prototype distortion task

**Fig. 2:**
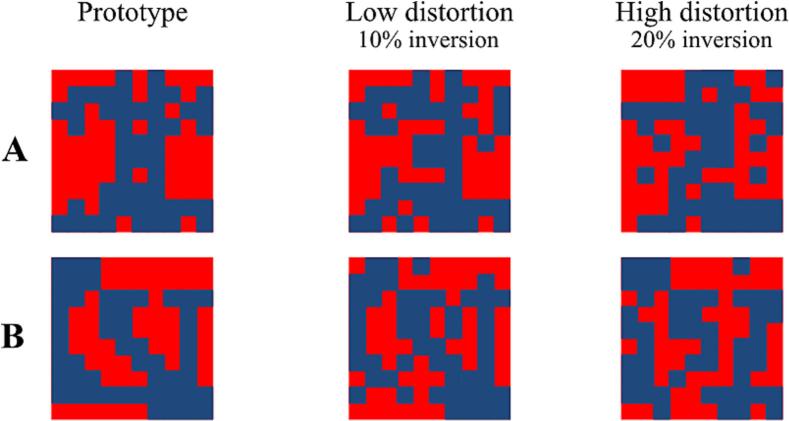
The A/B prototype distortion task. Stimuli (10 × 10 grids filled with red and blue squares) used in the prototype-distortion task were based on Seger et al. (2000) and Fried and Holyoak (1984). The prototypes (A,B) are shown on the left. Examples of low- and high distortion patterns (derived from the prototypes by inverting 10 % or 20 % of the squares) are illustrated on the right. (For interpretation of the references to color in this figure legend, the reader is referred to the web version of this article.)

The ‘A/B’ version of the prototype distortion task was used to assess categorization performance. The prototype stimuli were adapted from Seger et al. (2000), who based their investigation on the work of Fried and Holyoak (1984). Each stimulus consisted of a 10 × 10 grid filled with red and blue squares. Low and high distortion stimuli have been created by the random inversion of 10 % and 20 % of the prototype patterns (Fig. 2).

Participants were informed of the existence of the categories before the onset of the experiment and were required to learn the distinction between these categories through trial and error. This allowed us to additionally observe the process of category learning directly during the acquisition phase.

During the acquisition phase (training), subjects were exposed to the low and high distortion versions of the prototype, while during test and retest, low distortion, high distortion, and prototype patterns were all presented.

The instructions were explained to the subjects in written form. Before the beginning of the training phase, the participants were informed that they were going to learn to categorize paintings by two abstract artists, Smith and Jones, and that at the beginning, they would have to rely on guessing, but that they were going to receive continuous feedback on their performance.

The training phase consisted of ten blocks. In each block, 10 low distortion (5 derived from prototype A, 5 derived from prototype B) and 10 high distortion (5 from prototype A, 5 from prototype B) patterns were presented to the participants, each for 2500 ms. The participants were instructed to place each presented pattern into one of the categories by pressing either the left or the right arrow button on the keyboard. The time to respond was not limited. The program gave immediate feedback after the participant had made a decision, indicating whether or not the decision was correct. This message was displayed on the screen for 500 ms. The next pattern was presented 500 ms afterwards. The training phase was followed by an unfilled delay of 10 min.

The test phase consisted of four blocks. Ten low and ten high distortion patterns (form category A and B, 4 × 5) and the two prototypes (A and B) were presented in each block. The participants had to make the same category judgments as in the training phase, but no feedback about the accuracy was given this time. The task in the morning retest phase was identical to the evening test phase in every respect.

The mean number of correct responses was calculated for each subject individually for all 18 blocks (the 10 blocks of training /T1-T10/, the 4 blocks of the evening test /E1-E4/ and the 4 blocks of the morning retest /M1-M4/ sessions). Percentages of correct responses of Blocks T1 − T5, T6 − T10, E1- E4 and M1- M4 formed the values for the early and late training, and the evening and morning test phases, respectively.

We also monitored the alertness of the participants before the evening test and morning retest phase using a one-to-ten self-rated scale ranging from almost asleep (0) to fully awake (10).

### 2.5 BDNF genotyping

The procedure as described in Antal et al. (2010) for BDNF genotyping was followed. DNA was extracted using standard procedures from whole blood taken from the participants into ethylenediaminetetraacetic acid tubes. Primer sequences and polymerase chain reaction (PCR) conditions are available on request. The success of the PCR was checked for on agarose/2 × Tris/Borate/EDTA (TBE) gels. The restriction fragment length polymorphism (RFLP) analysis was performed by digesting the PCR product with the restriction enzyme Hsp92II. Restriction products were electrophoresed on a 2 % agarose gel and visualized using a transilluminator and ethidium bromide staining. After digestion, the *Val* allele (Guanine) produced two fragments, 57 and 217 bp, whereas the *Met* allele (Adenine) formed three, 57, 77, and 140 bp.

### 2.6 Statistical analysis

As this was the first study studying the interaction of tDCS and BDNF genotype on categorization, we could not compute our sample size based on the effect sizes of prior studies. Instead, we conducted a statistical power analysis using the G*Power 3.1 software, assuming a moderate effect size of 0.25, statistical power of 0.95, α error rate of 0.05, and correlation among repeated measures of 0.50. The analysis indicated a minimum required sample size of 44 participants. Given the positive findings of our previous study (Ambrus et al., 2011), which included 60 participants; we aimed for a sample size comprising a minimum of 60 healthy individuals, while meeting the parameters of the sample size calculation.

To ensure that the two groups were comparable at pre-test phase, we conducted a three-factorial analysis of variance (ANOVA) with the independent factors SEX (male and female), STIMULATION (anodal tDCS and sham) and BDNF GENOTYPE (*Val/Val* and *Met* carrier) for age and alertness both in the evening and on the following morning. We also tested whether the sample, based on stimulation received and BDNF genotype, was sex-matched using a Chi squared (χ^2^) test.

To check whether the baseline category learning was the same in the tDCS subgroups and in the BDNF genotype subgroups, a three-way ANOVA was conducted on the categorization performance at the early training phase with independent factors STIMULATION, SEX, and BDNF GENOTYPE. We implemented a four-way mixed model ANOVA test with mean category performance as the dependent variable, PHASE (early training (ET), late training (LT), evening test (Evn), and morning retest (Mor)) as the repeated factor, and STIMULATION, SEX, and BDNF GENOTYPE as independent factors to investigate the interaction between tDCS and BDNF polymorphism on category performance. We conducted Bonferroni-corrected pairwise comparisons for statistically significant findings. Effect sizes were computed as eta squared η^2^ and were interpreted corresponding to Cohen’s benchmark (small, η^2^ = 0.01; medium, η^2^ = 0.06 and large, η^2^ = 0.14) (Cohen, 1988). As the prototype items were not presented during the training phase, only responses for the high- and low distortion items were used for the time course analyses.

Statistical analysis of the data was conducted using IBM SPSS 28.0. Normality of the raw data or residuals was tested by visually verifying normality plots and using the Shapiro-Wilk test to meet the assumptions for parametric tests. For the ANOVAs, sphericity for the repeated factor was tested using Mauchly’s test of sphericity and in case of violations, Greenhouse-Geisser corrections of the degrees of freedom were applied. For the independent factor in the MANOVA, the homoscedasticity assumption was checked for using Levene’s test. All comparisons were two-tailed and were conducted with a significance level of p < 0.05.

## 3 Results

### 3.1 Participants

Sixty-two healthy adults, all students at the University of Göttingen, participated in the study (age: 24.7, SD: ± 3.80, see Table 1). The visual acuities of the participants were normal or corrected to normal. 31 participants (50 %) were women. Thirty-three participants (17 males) received the anodal tDCS stimulation, while 29 (14 men) received sham stimulation during the training phase of the task. The 62 participants were genotyped as follows: 38 participants were found to be homozygous for the *Val* allele (*Val66Val*), 23 were *Val66Met* heterozygotes, and 1 subject was homozygous for the *Met* allele. Following established procedures when the number of *Met* homozygotes is low, the *Val66Met* heterozygotes and the *Met66Met* subject were grouped into a single category (*Met* carriers) (Freundlieb et al., 2012, Soliman et al., 2010). The groups divided based on either BDNF profile, type of stimulation received or sex were all comparable for age and pre-test alertness at both time points (Table 1). At the early training phase, there were no significant differences in categorization performance either between the anodal and sham tDCS groups (F(1,54) = 0.0540, p = 0.817) or between the *Val/Val* and *Met* carrier groups (F(1,54) = 0.907, p = 0.345).

**Table 1:** Participant demographics and outcomes. Participant demographics and outcomes. A. Descriptive statistics of the sample. B. Three-factorial ANOVA for the demographic measures, wakefulness and categorization performance during early training phase (ET) with STIM (anodal tDCS, sham), SEX (male, female), and BDNF genotype (*Val/Val*, *Met carrier*) as categorical predictors. SD, standard deviation; STIM, stimulation group; BDNF, brain-derived neurotrophic factors.

A				Age (years)	Alertness Evening	Alertness Morning	Categorization performance at ET
Stimulationgroup	BDNF genotype	Sex	n	Mean SD	Mean SD	Mean SD	Mean SD
AnodaltDCS	*Val/Val*	Female	10	24.2 ± 4.23	6.38 ± 1.69	6.13 ± 2.10	67.7 ± 17.1
		Male	10	26.0 ± 3.38	6.88 ± 1.13	5.38 ± 1.19	66.0 ± 15.8
	*Met carrier*	Female	6	23.8 ± 3.70	7.20 ± 1.64	6.00 ± 1.22	69.5 ± 9.36
		Male	7	25.0 ± 7.55	6.33 ± 1.15	6.67 ± 1.53	67.4 ± 16.3

Sham	*Val/Val*	Female	10	22.5 ± 1.87	6.00 ± 2.10	5.00 ± 2.37	62.4 ± 13.0
		Male	8	25.5 ± 5.69	6.50 ± 1.73	6.50 ± 2.38	57.4 ± 7.17
	*Met carrier*	Female	5	24.1 ± 2.93	6.83 ± 0.98	6.50 ± 1.22	62.8 ± 15.6
		Male	6	26.5 ± 3.02	6.00 ± 1.67	5.17 ± 1.47	70.0 ± 14.4
Total			62	24.7 ± 3.80	6.52 ± 1.49	5.83 ± 1.70	65.5 ± 14.0

		Age performance		Alertness – Evening		Alertness – Morning		Categorization performance	
	df	F	*P*	F	*p*	F	*p*	F	*p*
STIM	1, 56	0.006	0.937	0.566	0.456	0.216	0.645	1.15	0.289
SEX	1, 56	2.95	0.0942	0.132	0.718	0.00150	0.969	0.0540	0.817
BDNF	1, 56	0.064	0.802	0.102	0.751	0.384	0.539	0.907	0.345
STIM×SEX	1, 56	0.244	0.624	0.000300	0.986	0.0135	0.908	0.0720	0.789
STIM×BDNF	1, 56	0.728	0.399	0.000700	0.979	0.216	0.645	0.275	0.602
SEX×BDNF	1, 56	0.0640	0.802	1.963	0.169	0.433	0.514	0.833	0.366
STIM×SEX× BDNF	1, 56	0.00100	0.981	0.000300	0.986	3.90	0.0556	0.925	0.340

### 3.2 Categorization performance

**Fig. 3:**
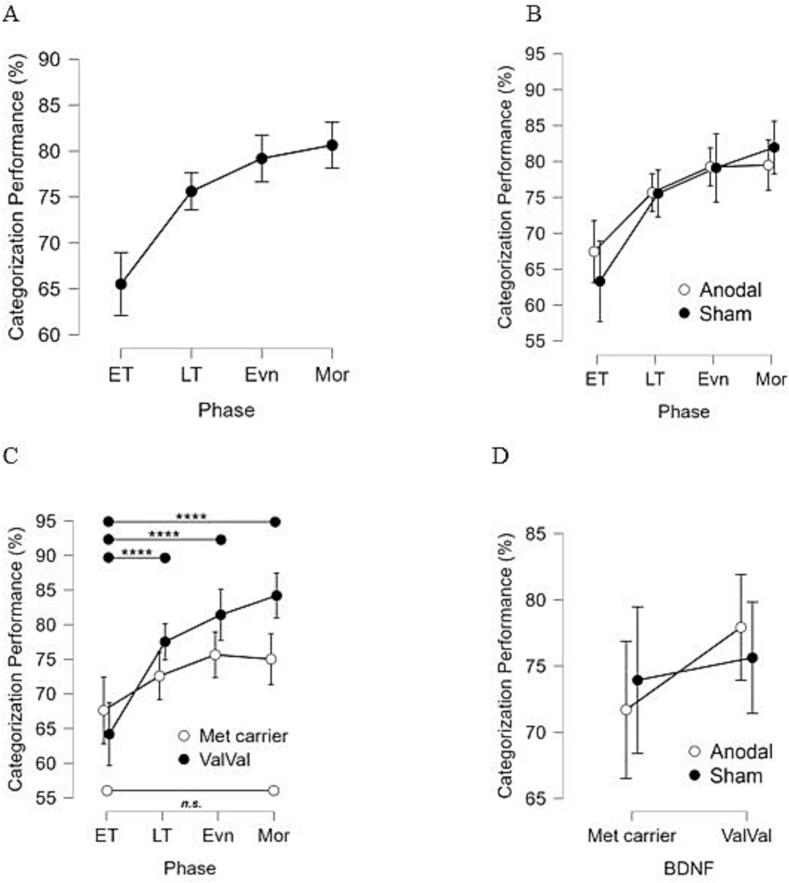
Categorization performance between groups over time. A. Exploration of main effect of phase on categorization performance. B. Changes in performance between sham and anodal tDCS groups over time. C. Changes in performance between *Met* carriers and *Val/Val* homozygotes over time, independent of stimulation type received. Higher performance was observed at later time points compared to early training only in *Val/Val* carriers, but no difference in performance across time for *Met* carriers. D. Changes in performance between sham and anodal tDCS groups comparing *Met* and *Val/Val* carriers. ET = early training, LT = late training, Evn = evening test, Mor = morning retest. **** *p* < 0.0001, Bonferroni. Error bars denote ± standard error of the mean.

The MANOVA showed a large significant main effect of PHASE (Table 2, p < 0.001, η_p_^2^ = 0.294), with higher performances across later phases compared to early training (ET) phase (all pairwise comparisons to ET phase at p < 0.0001) (Fig. 3A). No substantial improvement or decline in performance was observed among the late training, evening, and morning test phases (all pairwise comparisons at p > 0.12, Fig. 3A). The main effect of PHASE demonstrated learning over the early training phase with no additional significant gain in performance afterwards. Addressing the impact of BDNF genotype on tDCS effects on category learning using the A/B prototype distortion task, no significant interaction among PHASE, STIM and BDNF GENOTYPE was observed (F_G-G_(2.03,110) = 0.659, p = 0.522, Table 2). We did not find any significant differences in performance between the anodal tDCS and sham group over time as shown by the non-statistically significant interaction between STIMULATION and PHASE (Fig. 3B).

**Table 2:** Mixed Model ANOVA for the categorization performance with PHASE (early and late training, evening test, morning retest) as within-subject factor, and STIM (anodal tDCS, sham), SEX (male, female), and BDNF genotype (*Val/Val*, *Met carrier*) as categorical predictors. Values in bold represent significant main effect or interactions (p < 0.05), _G-G_ represents analyses with Greenhouse-Geisser corrections (ε = 0.678) where Mauchly's test indicated that the assumption of sphericity had been violated (χ^2^[5] = 35.7, p < 0.01).

Effects on categorization performance	df	F	*p*	η_p_^2^
STIM	1, 54	0.000	0.986	—
SEX	1, 54	0.0710	0.791	—
BDNF	1, 54	1.02	0.317	—
STIM × Sex	1, 54	0.170	0.681	—
STIM × BDNF	1, 54	0.317	0.576	—
Sex × BDNF	1, 54	0.682	0.412	—
STIM × SEX × BDNF	1, 54	0.104	0.748	—
PHASE _G-G_	2.03, 110	22.5	<0.001	0.294
PHASE × STIM _G-G_	2.03, 110	1.07	0.348	—
PHASE × SEX _G-G_	2.03, 110	1.03	0.361	—
PHASE × BDNF _G-G_	2.03, 110	4.09	0.0190	0.0700
PHASE × STIM × SEX _G-G_	2.03, 110	0.656	0.547	—
PHASE × STIM × BDNF _G-G_	2.03, 110	0.659	0.522	—
PHASE × SEX × BDNF _G-G_	2.03, 110	0.0310	0.971	—
PHASE × STIM × SEX × BDNF _G-G_	2.03, 110	1.94	0.148	—

There was a medium-sized significant interaction between the BDNF GENOTYPE and PHASE (Table 2, p = 0.0190, η_p_^2^ = 0.0700) with *Val/Val* participants demonstrating substantial learning compared to the early training phase (n = 38, all pairwise comparisons at p < 0.0001). No further significant change in performance was observed when comparing phases other than the early training (all pairwise comparisons at p > 0.05) (Fig. 3C).

We could not find a significant interaction between STIMULATION and BDNF GENOTYPE (Fig. 3D).

## 4 Discussion

The main objective of this study was to test how BDNF polymorphism status impacts the potential enhancing effects of anodal tDCS over the right DLPFC at a current of 1 mA for 10 min on category learning. In contrast to our hypothesis, we found no evidence for the effect of tDCS, when using our specific stimulation parameters, on categorization performance in general, or for its elevating effect on *Val/Val* homozygote or *Met* carrier performance. Nevertheless, testing the polymorphism’s effect on categorization performance, we have found that the performance of the *Val/Val* subjects improved significantly along the time course of the task, but not that of *Met* carriers.

Previous studies have demonstrated that modulating the prefrontal cortex activity by anodal tDCS is associated with enhanced cognitive ability; for example, better cognitive control (Plewnia et al., 2015), enhanced working memory performance (Brunoni and Vanderhasselt, 2014), improved planning activities (Dockery et al., 2009), as well as category learning (Ambrus et al., 2011, Clark et al., 2012, Coffman et al., 2012, Gibson et al., 2020). Our findings failed to support the potential of anodal tDCS over the DLPFC to enhance cognition. Our non-conclusive results on the impact of tDCS on category learning contradicts our previous findings. In Ambrus et al. (2011), the same stimulation parameters were implemented as in the current study and it was shown that DLPFC-tDCS modulated categorization performance in healthy participants. However, in that study, the A/not A variant rather than A/B variant of the prototype distortion task was used. Our lack of improvement in categorization was similar to Verhage et al. (2017), which implemented 20 mins of anodal tDCS at 1.5 mA over the right cerebellum and a rule-based categorical task. The variability of the above findings across the different studies potentially arises from the heterogeneous nature of stimulation parameters, site of stimulation, and type of category learning task used across the different studies investigating category learning. It has been demonstrated that the electrode position and size, the intensity of the current, the stimulation duration as well as the underlying brain state may significantly alter the cortical effects induced by tDCS (Nitsche et al., 2003, Vignaud et al., 2018, Woods et al., 2016). With regards to the stimulation parameters implemented in our current study, they might not be optimal to induce the desired plasticity. In a recent study by Farnad et al. (2021) exploring the neuroplastic effects of anodal tDCS of the M1 using different intensities and stimulation durations, it was found that stimulation at 1 mA for 15 min (same current density at anode as our current study) led to the smallest increase in cortical excitability compared to higher intensities and durations. Increasing the stimulation intensity to 3 mA and increasing the duration to 20 or 30 min stimulation might lead to better neuroplastic changes. Nevertheless, it is important to note that Farnad et al. (2021) included only older adults ranging from 50 to 80 years of age were included while in our study, most participants were young adults. Furthermore, applying tDCS during a task might need different intensities (Antal et al., 2007, Paulus and Rothwell, 2016). Combining anodal tDCS at 1 mA for 10 min with a cognitive task has been shown to reduce cortical excitability compared to tDCS alone, denoting reduced tDCS-induced plasticity (Antal et al., 2007). One future consideration might be to use homeostatic stimulation to downregulate the target area prior to combination (Lang et al., 2004).

In respect to the methods to study category learning, four tasks have been most commonly used in the field − prototype distortion tasks, rule-based tasks, information-integration tasks, and the weather prediction task. It has been shown that the cognitive, neuropsychological, and imaging findings significantly differ depending on the type of task used (Ashby and Maddox, 2005, Ashby and Maddox, 2011). The *A/B* prototype distortion task has been shown to differ in the brain areas activated during the task compared to the A/notA task (Seger et al., 2000, Zeithamova et al., 2008).

One of the major contributing factors to the high interindividual variability in tDCS task- and activity-dependent effects is the genetic makeup (Veronica Witte et al., 2012), and BDNF stands out as an excellent candidate gene for influencing the effects of non-invasive brain stimulation (Wiegand et al., 2016). It has been shown that *Val66Met* carriers exhibit larger motor cortical excitability after anodal tDCS over the M1 and larger cortical inhibition after cathodal tDCS compared to *Val/Val* homozygotes, as measured by transcranial magnetic stimulation (Antal et al., 2010). Another study by Puri et al. (2015) demonstrated that older adults carrying the *Met* allele showed higher corticospinal excitability after 20 min of anodal M1-tDCS compared to homozygotes. Recently, a study tested how *Val66Met* polymorphism modulates brain activity after repetitive TMS, which was delivered over the left frontal cortex during a visual memory task (Abellaneda-Pérez et al., 2022). They found that only *Val/Val* carriers but not *Met* carriers exhibited reduced memory performance after rTMS delivered to the left frontal cortex compared to vertex stimulation (control condition). However, the association between tDCS and BDNF polymorphism on cognition still remains elusive. In our study, in which we tried to elucidate such influence, the lack of significant interaction among phase, BDNF genotype, and anodal tDCS over DLPFC contradicts the above discussed findings. This lack of influence of BDNF polymorphism on tDCS effects on category learning using a A/B prototype distortion task can first be explained by the lack of significant differences in performance between the active and sham tDCS groups.

Our study had a considerable sample size and showed no baseline differences in the categorization performance between either anodal and sham tDCS groups or *Met* carriers and *Val/Val* homozygotes. Our results show the inability of DLPFC tDCS at 1 mA for 10 min to change cortical excitability to alter category learning. They also show that BDNF polymorphism does not influence tDCS effects on categorization performance.

Nevertheless, we found that, over time, *Met* carriers failed to show a category learning effect, which was prominent in the *Val/Val* homozygotes. This finding is in line with the results of previous studies supporting that *Met* carriers perform worse on episodic memory tasks than *Val/Val* homozygotes (Cathomas et al., 2010, Dempster et al., 2005, Egan et al., 2003, Goldberg et al., 2008, Hariri et al., 2003), with which category learning is associated (Tunney and Fernie, 2012). In comparison to Abellaneda-Pérez et al. (2022), who found that individuals with *Val/Val* polymorphism exhibit higher baseline performance on a visual memory task than *Met* carriers, we did not encoutner any difference in categorization performance at the early training phase between the groups. In addition, *Val/Val* homozygotes seemed to show a consolidation of the learned categories by way of their even better test results in the morning test phase while the hit rate of *Met* carriers declined, supporting the work of Rovný et al. (2023).

Our findings must be interpreted in the light of the study limitations. In our study, only one cortical area has been targeted by tDCS. While the DLPFC plays a major role in category learning, other brain areas such as the ventromedial prefrontal cortex and anterior hippocampus (Bowman et al., 2020) as well as the fusiform gyrus (Folstein et al., 2012) have been identified being involved in prototype-based category learning. Future studies might implement multifocal tDCS (Gregoret et al., 2021, Ruffini et al., 2014) simultaneously to different cortical areas, for instance the DLPFC and ventromedial prefrontal cortex, potentially leading to larger impact on prototype-based category learning. Moreover, temporal interference stimulation holds promise for targeting deep brain areas involved in categorization such as the hippocampus and fusiform gyrus (Modak et al., 2024, Violante et al., 2023). Furthermore, we examined only a single tDCS protocol—1 mA for 10  min with a fixed electrode montage—which limits the generalizability of our findings regarding the broader efficacy of optimal stimulation parameters. In our study, the sample of *Met* carriers included only one *Met/Met* homozygote. Conducting the BDNF genotype analysis while combining the homozygote and the *Val/Met* carriers might have obscured more subtle allele-specific effects. Nevertheless, it is important to note that around 12 % of *Met* carriers in healthy European populations tend to be homozygotes, while the rest are *Val/Met* carriers (Vulturar et al., 2016). This observed prevalence rate is close to what is observed in our sample. Moreover, deterministic feedback is used during the training phase. The implementation of probabilistic feedback through the probabilistic prototype distortion task offers increased flexibility in manipulating the probability of category membership and randomness within the task, making the study of category learning more realistic (Marchant and Chaigneau, 2021). Future studies should implement edge-based (i.e. contour/shape) features rather than surface-based features (i.e. texture/color) when implementing the prototype distortion task, as it has been shown to enhance the reliability and sensitivity of the task for detecting category-learning effects (Zhou et al., 2019).

## 5 Declaration of generative AI scientific writing

During the preparation of this work, no AI and AI-assisted technologies have been used to write the manuscript. The work presented has been written by the authors.

## Funding

The study was conducted without any funding.

## Declaration of competing interest

The authors declare the following financial interests/personal relationships which may be considered as potential competing interests: PR is supported by EU-Horizon 2020 (PAINLESS) and has received honorarium from Neurocare (Germany) for a teaching course. AA has received consulting fees from Neurocare (Germany) and Elsevier, she is a paid advisor by EPI (USA). AA is supported by German Research Foundation, DFG (AN 687/9-1, VIRON) and EU-Horizon 2020 (PAINLESS). However, none of these competing interests could have appeared to influence the work reported in this paper. Other co-authors have no competing interests to declare.
